# Pathogenic Mechanisms Associated With Different Clinical Courses of Multiple Sclerosis

**DOI:** 10.3389/fimmu.2018.03116

**Published:** 2019-01-10

**Authors:** Hans Lassmann

**Affiliations:** Center for Brain Research, Medical University of Vienna, Vienna, Austria

**Keywords:** relapsing remitting MS, secondary progressive MS, primary progressive MS, inflammation, demyelination, neurodegeneration

## Abstract

In the majority of patients multiple sclerosis starts with a relapsing remitting course (RRMS), which may at later times transform into secondary progressive disease (SPMS). In a minority of patients the relapsing remitting disease is skipped and the patients show progression from the onset (primary progressive MS, PPMS). Evidence obtained so far indicate major differences between RRMS and progressive MS, but no essential differences between SPMS and PPMS, with the exception of a lower incidence in the global load of focal white matter lesions and in particular in the presence of classical active plaques in PPMS. We suggest that in MS patients two types of inflammation occur, which develop in parallel but partially independent from each other. The first is the focal bulk invasion of T- and B-lymphocytes with profound blood brain barrier leakage, which predominately affects the white matter, and which gives rise to classical active demyelinated plaques. The other type of inflammation is a slow accumulation of T-cells and B-cells in the absence of major blood brain barrier damage in the connective tissue spaces of the brain, such as the meninges and the large perivascular Virchow Robin spaces, where they may form aggregates or in most severe cases structures in part resembling tertiary lymph follicles. This type of inflammation is associated with the formation of subpial demyelinated lesions in the cerebral and cerebellar cortex, with slow expansion of pre-existing lesions in the white matter and with diffuse neurodegeneration in the normal appearing white or gray matter. The first type of inflammation dominates in acute and relapsing MS. The second type of inflammation is already present in early stages of MS, but gradually increases with disease duration and patient age. It is suggested that CD8^+^ T-lymphocytes remain in the brain and spinal cord as tissue resident cells, which may focally propagate neuroinflammation, when they re-encounter their cognate antigen. B-lymphocytes may propagate demyelination and neurodegeneration, most likely by producing soluble neurotoxic factors. Whether lymphocytes within the brain tissue of MS lesions have also regulatory functions is presently unknown. Key open questions in MS research are the identification of the target antigen recognized by tissue resident CD8^+^ T-cells and B-cells and the molecular nature of the soluble inflammatory mediators, which may trigger tissue damage.

## Introduction

Multiple sclerosis is a chronic inflammatory disease of the central nervous system which leads to the formation of focal confluent lesions of primary demyelination in the white and gray matter and to diffuse damage and neurodegeneration in the entire brain (1). In general the disease starts in patients in the third decade of life with a relapsing and remitting clinical course. On average after 10–15 years the disease in the majority of patients converts into a course of slow progression (secondary progressive MS). In a subset of patients, in particular in those with higher age at onset, the disease starts with a progressive course [primary progressive MS; (2)]. It is currently an open debate, whether primary progressive MS is a distinct disease entity or whether it just represents part of the variable clinical disease spectrum (3–5). This question has major pathogenic implications. Most researchers regard MS as a primary inflammatory disease, in which demyelination and tissue injury is driven by immune mediated mechanisms throughout all different stages and in all different courses (6, 7). In this case PPMS would be just a clinical variant of a common disease process. The other view suggests that MS is a primary neurodegenerative disease, which is modified and amplified by the inflammatory process. In this situation PPMS could reflect the primary disease process of MS and the other courses (RRMS and SPMS) are those, modified by an inflammatory reaction (3).

There is no doubt that major differences exist between the relapsing and progressive stages of MS and this is also reflected by the different response to currently available immunosuppressive or immunomodulatory treatments (8, 9). However, there is an overlap in pathological features, pathogenic mechanisms and therapeutic responses between relapsing and progressive MS (10, 11). In particular, evidence for subclinical disease activity, defined by the presence of new focal contrast enhancing lesions, can be present in patients with SPMS as well as PPMS. For this reason, it has been suggested to classify MS patients, who have entered the progressive disease stage into those with or without evidence of disease activity and with or without disease progression (2). The consequence of such a clinical disease classification could be to skip the distinction between primary and secondary progressive MS. Whether this may be justified or not and what are the pathogenic implications will be discussed in this review article.

## Clinical Course, Epidemiology, and Genetics

### Clinical and MRI Features

The term primary progressive MS clinically defines a disease, which develops with increase of neurological deficits in the absence of prior or intermittent exacerbations and remissions. This differs from the relapsing-remitting course of the disease, characterized by new bouts of the disease followed by stages of clinical remission. Relapsing/remitting MS may after several years of disease duration, and when patients have reached a moderate level of clinical disability (EDSS scape 3–4), transform into a secondary progressive disease course (12, 13). While disease relapses are associated with new and contrast enhancing lesions in MRI, the brain and spinal cord changes during progressive disease were thought to be reflected by a steady increase of brain and spinal cord atrophy. However, using more sophisticated tools for clinical monitoring of the patients, as for instance applied in controlled therapeutic trials, it turned out that a significant proportion of patients with PPMS and SPMS show signs of clinical or MRI-based “disease activity” (2) as defined above. Overall, no qualitative differences regarding disease activity between PPMS and SPMS were found, although, as reflected by the original disease definitions, relapses associated with new focal white matter lesions are less frequent in PPMS. Similarly, no essential differences between SPMS and PPMS were seen by MRI (14).

The average disease onset in patients with RRMS is within the third decade of life. In contrast disease onset in patients with PPMS peaks in the 5th decade of life, which is similar to the age, when patients with RRMS tend to convert into SPMS (15, 16). Clinical disease severity and the speed of disease progression is highly variable between patients, but on average the speed of progression is similar between patients with PPMS and SPMS, and is independent from the severity of previous relapses of the disease (12, 13).

## Genetics

The male to female ratio in patients with RRMS and SPMS is 1:3, while patients with PPMS show a lower female predominance (10, 17). Interestingly, disease risk is also transferred from unaffected females to their MS affected offspring than from males, raising the possibilities of the involvement of mitochondrial genes, epigenetic effects or a pathogenic role of intrauterine exposure to exogenous risk factors (18). Genome wide association studies have now identified numerous gene regions, associated with increased disease susceptibility, each of the individual genes providing only a very minor effect (19, 20). Interestingly nearly all of the gene regions identified so far contain genes involved in immune mechanisms, which is in line with clinical, immunological, and neuropathological data defining MS as an immune mediated disease. Importantly, within the familial risk in multiplex families there is no clear discrimination between the different MS courses. Thus, within the same family different patients may develop relapsing, secondary or primary progressive MS, although the concordance rate of clinical courses is moderately increased in siblings of the PPMS cohort (21, 22). In line with these observations, so far no clear differences in genetic associations became evident between PPMS and other disease forms in genome wide association studies (23). However, recent studies suggest that different genes may be associated with relapse risk vs. the speed of EDSS increase (24) and genetic variants, described to be pathogenic in some neurodegenerative diseases, have been identified in a (small) subset of patients with PPMS, but not in patients with other disease courses (23). One of these examples is a variant of a gene involved in transcriptional regulation (NR1H3), which was only found to be associated with PPMS, but not with other disease forms (25). This observation, however, also highlights a caveat regarding the interpretation of such data, since it has not been confirmed in a detailed analysis of the much larger dataset (26). Overall, however, these data indicate that there is a basic polygenic pattern determining the global MS risk and this is the same for all disease courses and involves immune mediated mechanisms (27), while the development of progressive disease may be additionally fostered by genetic variants associated with lipid metabolism or neurodegeneration. However, this may not apply for all, but only for a small subset of patients with PPMS.

## Immunology and Biomarkers

Many immunological studies have been performed with the aim to identify MS specific biomarkers and disease mechanisms and to find markers able to predict clinical disease course and outcome. These data are summarized in comprehensive recent review articles (28, 29). Besides MRI and markers related to therapy (induction of blocking antibodies) or JC virus infection, they include markers for neurodegeneration, such as neurofilaments, markers for astrocytic activation (e.g., chitinase or GFAP). Neurofilament protein detected in the serum or cerebrospinal fluid appears to be a good marker for the extent of active neurodegeneration, but this is not MS specific. Chitinase may be a good marker for active disease in relapsing remitting disease, reflecting the degree of astrocyte activation, or damage in active lesions.

So far the highest clinical relevance is reported for the presence of intrathecal immunoglobulin synthesis, reflected by an increased IgG index and oligoclonal bands. It is associated with MS with high sensitivity, but found also in other (chronic) inflammatory diseases of the central nervous system (30). Regarding PPMS its presence is an important paraclinical marker for diagnosis and, thus, detection of intrathecal IgG synthesis has been re-introduced into the new diagnostic criteria (31). Cytokines, chemokines, and adhesion molecules have been analyzed and a comprehensive immunophenotyping of inflammatory cells in the cerebrospinal fluid has been performed as well. Overall these studies showed increased levels in MS serum and CSF, being most significantly altered in patients with (active) RRMS followed by patients with SPMS and PPMS (32–34). These markers have some clinical value for diagnosis and monitoring of disease activity, but none of them have turned out to specific for MS. In addition, so far no specific serum or CSF marker profile has been identified, which allows the distinction between SPMS and PPMS.

To overcome this problem, the question regarding potential biomarkers for MS diagnosis and clinical subtypes has recently been approached with an innovative technology. By using an unbiased simultaneous screening for the concentration of 1.128 proteins together with new machine learning and bioinformatics technology, CSF protein profiles were established in a large sample of patients with RRMS, SPMS, and PPMS and the findings were compared with those seen in patients with other inflammatory and non-inflammatory CNS diseases (35). Using these new tools profiles were detected, which allowed to differentiate between MS and other inflammatory or non-inflammatory CNS diseases and to clearly separate RRMS from progressive forms of the disease. However, no significant differences appeared in the comparison between SPMS and PPMS. Deciphering the biomarker profile defined important pathogenic pathways. The protein profiles, which allowed the best differentiation between MS and other inflammatory CNS diseases, were those related to B-cell and Plasma cell function. This may represent an independent confirmation of the long standing observation that intrathecal immunoglobulin synthesis occurs in MS patients (30, 36). However, it also is in line with observations from pathology, that the contribution of B-cells differentiates MS lesions from non-MS inflammatory brain diseases better than it is the case for T-cell subsets or the activation of macrophages and microglia (37, 38). However, this profound B-cell component in the inflammatory response may not be specific for MS, since it is apparently also seen in certain other chronic human inflammatory diseases of the central nervous system, such as neurotuberculosis, borreliosis, lues, and others (39–41).

The CSF protein profiles most significantly associated with progressive MS were related to the formation of tertiary lymph follicles, and these markers were also associated with the extent of subpial cortical demyelinating pathology (42). Other markers being prominent in patients with progressive disease were related to innate immunity activation and oxidative injury as well as markers, which reflect neuronal and axonal injury, such as for instance neurofilament protein (42–44).

In another approach an un-biased metabolomic plasma profiling has been performed in PP vs. RRMS patients and the data were further compared to those obtained from patients with Parkinson's disease and healthy controls (45). The most dramatic metabolic changes were seen in PPMS patients and were mainly related to decreased profiles of glycerophopholipids and linoleic acid metabolism. These changes were not only present in the global MS population in comparison to controls, but even allowed to discriminate PPMS from RRMS. SPMS patients were not included in this study. It remains unresolved, whether these lipid changes just reflect the higher degree of global demyelination and neurodegeneration in progressive MS vs. RRMS. In addition, information on these lipid changes in proper disease controls with brain inflammation, demyelination or neurodegeneration is very limited.

Overall the immunological and metabolic data suggest that there are quantitative differences in immunological and neurobiological marker profiles between relapsing and progressive MS, which indicate that inflammation (systemic and intrathecal) is more pronounced in patients with relapsing disease and neurodegenerative events are more severe in the progressive stage of the disease. However, such differences vanish, when SPMS and PPMS patients are directly compared.

## Neuropathology and Immunopathology

**Figure 1:**
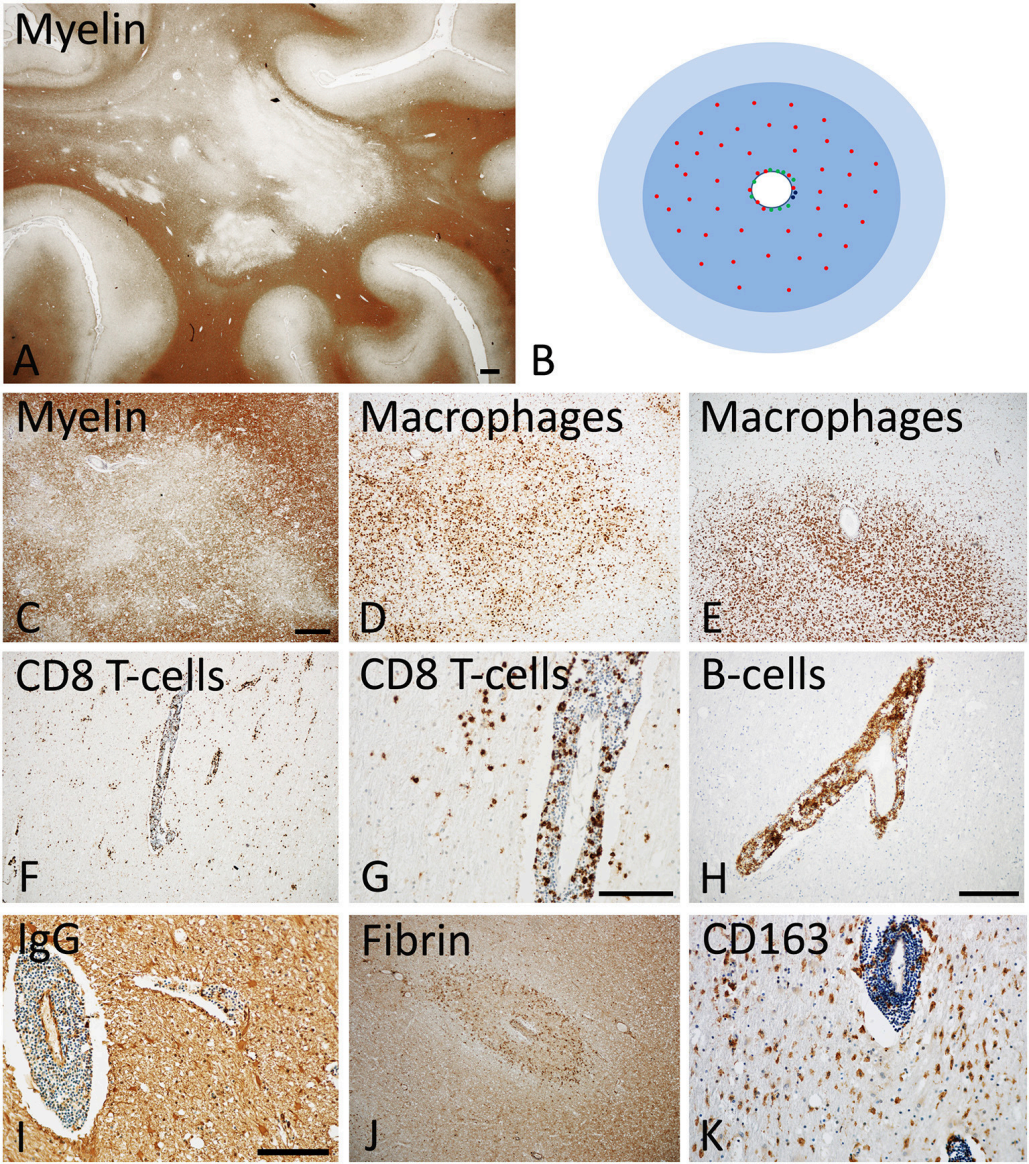
Active Lesions in early MS (acute and RRMS). **(A)** The dominant pathology in early MS is the presence of focal confluent demyelinated lesions in the white matter, many of them being in the stage of activity; section of a patient with acute multiple sclerosis, stained for myelin by immunohistochemistry for proteolipid protein. Magnification bar: 1 mm. **(B)** The classical active lesions in early MS develop around a central vein with inflammatory infiltrates, composed of CD8^+^ T-cells (red), CD20 positive B-cells (green), and few plasma cells (blue). While B-cells and plasma cells mainly remain in the perivascular space, the CD8^+^ T-cells also diffusely infiltrate the lesion parenchyme. The lesion (blue) is massively infiltrated by macrophages. Many of the lymphocytes are in the process of passing the vessel wall and this is associated with profound blood brain barrier leakage. This results in profound edema, which expands beyond the area of active demyelination (light blue). **(C–E)** Myelin staining (immunocytochemistry for proteolipid protein) shows patchy areas of active demyelination, which is associated with dense infiltration of the tissue by macrophages **(D,E)**. **(F, G)** Immunohistochemistry for the T-cell marker CD8 shows perivascular accumulation of T-cells, and their diffuse infiltration of the lesion parenchyme. **(H)** The perivascular inflammatory infiltrates contain numerous CD20^+^ B-lymphocytes. **(I,J)** Staining for IgG reveals massive leakage of the blood brain barrier and only a small number of IgG containing plasma cells in the perivascular space **(I)**; the profound blood brain barrier leakage is also reflected by extensive leakage of fibrinogen through the inflamed vessels **(J)**. **(K)** A subset of macrophages expresses the activation marker CD163, a feature which is typically found in active MS lesions. The magnification bars in the figures **(C,G,I)** represent 100 μm. Similar histological images as shown in this figure have been previously published. Structure of the lesions: Frischer et al. (46); Inflammatory reaction: Frischer et al. (47); Machado Santos et al. (37); Microglia and macrophages: Zrzavy et al. (38); Fibrin and blood brain barrier injury: Hochmeister et al. (48).

There are several pathological hallmarks, which distinguish MS from other diseases of the central nervous system (1). The most specific pathological changes are focal lesions with primary demyelination and astrocytic scaring, which develop on the background of a chronic inflammatory process (Figure 1). These lesions are not restricted to the white matter, but are also abundant in the gray matter of the cortex, the deep brain stem nuclei and the spinal cord (49–51). Primary demyelination means that myelin sheaths and their supporting cells, the oligodendrocytes, are destroyed, while axons are at least in part preserved. However, axonal and neuronal injury in gray and white matter lesions is pronounced. When it passes the threshold of functional compensation its extent is currently the best pathological predictor for permanent neurological deficit in the patients (52). Focal demyelinated lesions in the white and gray matter can be partly or completely repaired by remyelination, although the degree of remyelination is highly variable between patients (53, 54). In addition to these focal changes, diffuse neurodegeneration is present in the normal appearing white and gray matter, which results in brain atrophy, reflected by profound focal and diffuse loss of brain and spinal cord volume. All these changes are present in all MS patients, but their relative contribution to the global pathology varies between different patients and different forms, courses, or stages of the disease.

### Inflammation

**Figure 2:**
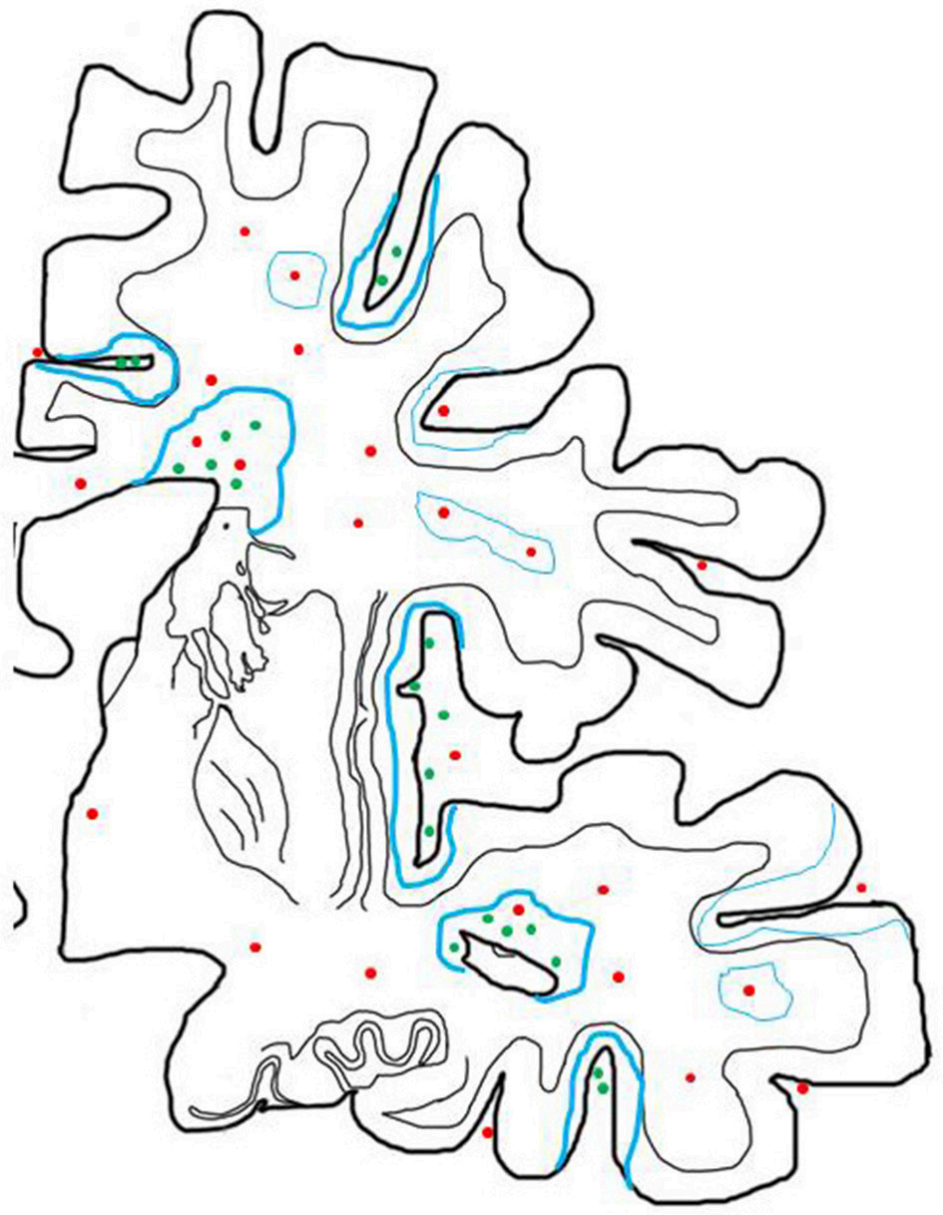
Inflammatory reaction in the brain of patients with progressive MS and its relation to active demyelination and neurodegeneration. The inflammatory reaction in the brain of patients with progressive MS is mainly seen in the large connective tissue spaces of the meninges and the periventricular Virchow Robin spaces. These inflammatory sites mainly contain CD8^+^ T-cells, a major component of CD20^+^ B-cells and a variable number of plasma cells and may in their most severe manifestations become organized in structures with features of tertiary lymph follicles (green dots). In addition there are perivascular cuffs mainly composed of CD8^+^ T-cells, which are more broadly dispersed within the white matter of the brain (red dots). Inflammation with T-cells, B-cells and Plasma cells (green dots) is associated with slow expansion of demyelinated lesions, defined by a rim of activated microglia cells, which in part contain early myelin degradation products in the cortex and the white matter (thick blue lines). Active demyelination and diffuse tissue injury occurs at a distance from the lymphocytic infiltrates and may, thus, be propagated by a soluble demyelinating or neurotoxic factor. Inactive plaques (thin green lines) can still be centered by a vein with a dominant infiltrate by CD8^+^ T-cells (red dots).

**Figure 3:**
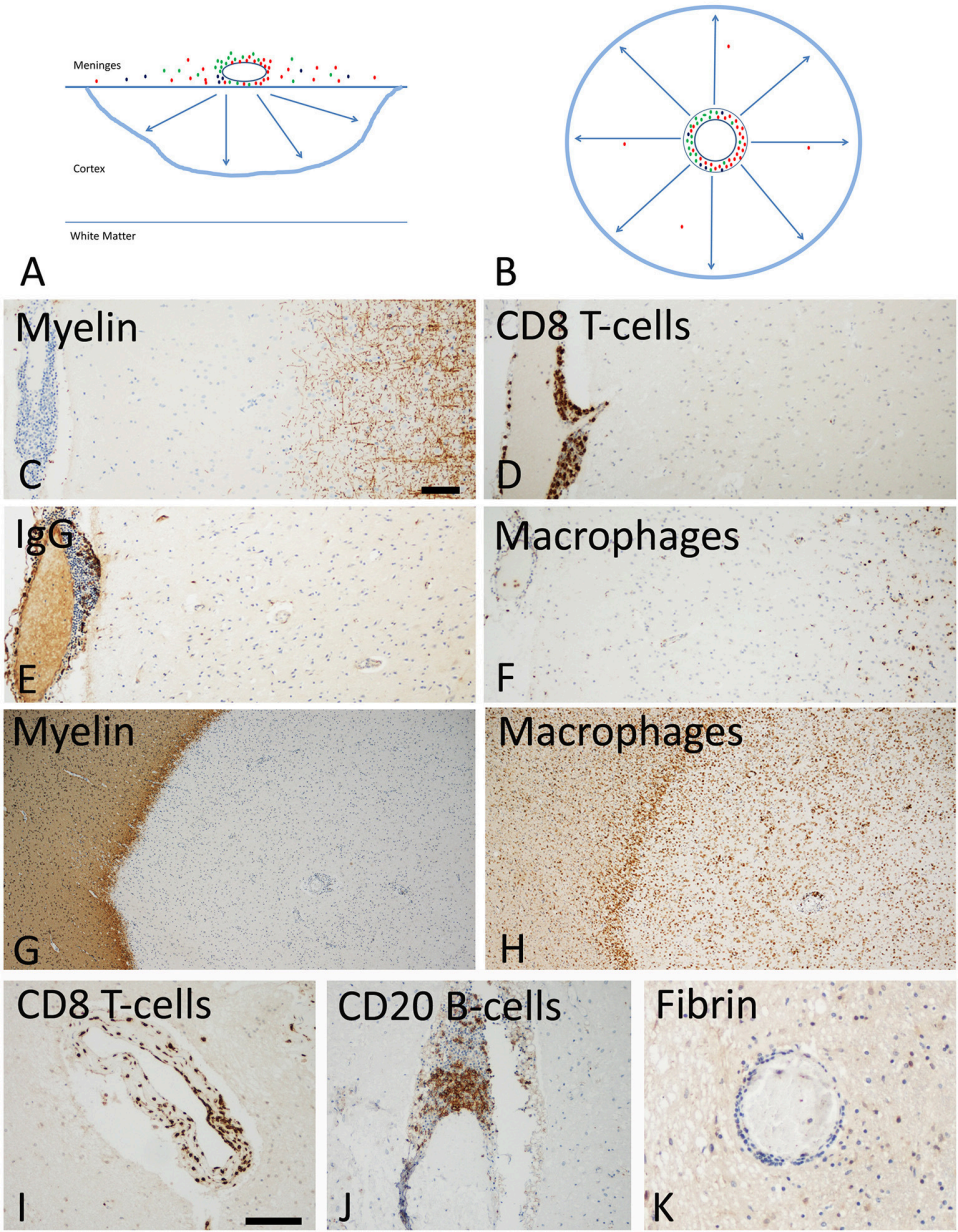
Slowly expanding lesions in the progressive stage of MS in the cortex and the white matter. **(A)** Active cortical lesions are associated with inflammatory infiltrates in the meninges, which are composed of CD8^+^ T-cells (red), CD20^+^ B-cells (green) and plasma cells (blue). Active demyelination occurs at a distance of the inflammatory infiltrates and is associated with activated microglia (blue lesion rim). The lesions gradually expand from the pial surface of the cortex toward the depth of the gray matter. Lymphocyte infiltrates are rare or completely absent in the cortical tissue and in particular at the zone of active demyelination. It is suggested that the inflammatory infiltrates in the meninges produce a soluble factor, which induces demyelination and neurodegeneration either directly or indirectly through microglia activation (arrows). **(B)** In slowly expanding lesions in the white matter T-cell, B-cell and plasma cell infiltrates are present in the large perivascular Virchow Robin spaces. Active demyelination and neurodegeneration occurs at a distance and is associated with microglia activation. Also in these lesions it is suggested that demyelination and neurodegeneration is driven by a soluble factor, produced by the perivascular lymphocytes or plasma cells (arrows). **(C–F)** Active cortical lesion in a patient with progressive MS. Subpial myelin is completely lost in an area with meningeal inflammation **(C)**; CD8^+^ T-cells are present in the meningeal infiltrates, but do not enter the cortical parenchyme **(D)**; The meningeal infiltrates also contain IgG positive plasma cells **(E)**, there is however no indication of IgG leakage from the vessels into the tissue, suggesting an intact blood brain barrier. Activated microglia and macrophages are seen at the site of active demyelination in the depth of the gray matter **(F)**. **(G,H)** Slowly expanding lesion in the white matter of a patient with progressive MS. The inactive plaque center contains vessels with perivascular cuffs of lymphocytes but the active demyelination at the lesion edge is associated with a rim of activated microglia **(G,H)**. Lymphocytes, such as for instance CD8^+^ T-cells and B-cells are present in the large perivascular space of the vessels, but there is little or no infiltration into the lesion parenchyme **(I,J)**. No fibrinogen leakage is observed around inflamed vessels, indicating intact blood brain barrier function **(K)**. Magnification bar representative for all images: 100 μm. Similar histological images as shown in this figure have been previously published. Structure of the lesions: Frischer et al. (46); Inflammatory reaction: Frischer et al. (47); Machado Santos et al. (37); Microglia and macrophages: Zrzavy et al. (38); Fibrin and blood brain barrier injury: Hochmeister et al. (48).

MS is a chronic inflammatory disease of the central nervous. Inflammation, characterized by the presence of perivascular T- and B-lymphocytes and their dispersion into the parenchyma, is most pronounced in patients, who have died in early stages after disease onset and declines with age of the patients and disease duration [(47); Figure 1]. However, even in the progressive stage of the disease pronounced inflammation is present, which is quantitatively in the range of other acute and chronic infectious or inflammatory diseases and massively exceeds that seen in patients with metabolic or neurodegenerative diseases (37). In progressive disease pronounced inflammation is mainly seen in those patients with clinical or radiological evidence of disease activity or of ongoing disease progression during the last months or year (Figures 2, 3), while in patients with stable disease during the last year prior to death and/or at very late disease stages tissue infiltration by leukocytes may decrease to levels present in age matched controls (47). In these patients ongoing active axonal injury, detected by focal accumulation of amyloid precursor protein as a marker for disturbance of fast axonal transport, has also declined to the levels seen in age matched controls (47). This adaptive inflammatory process is associated with microglia activation and infiltration of the tissue by macrophages, which is most extensive at sites of active demyelination and neurodegeneration, but, in particular in patients with progressive disease, diffusely affects also the normal appearing white and gray matter.

Similarly as in other chronic inflammatory diseases of the human CNS, inflammatory cells from the adaptive immune system mainly consist of MHC Class I restricted CD8^+^ T-cells, while MHC class II restricted CD4^+^ T-cells are rare or even absent [(55, 56); Figure 1]. These T-cells display the phenotype of resident memory cells and show focally restricted activation within active lesions (37, 57). It has been suggested from experience obtained in models of autoimmune encephalomyelitis that CD4^+^ T-cells are the major drivers of the inflammatory process, a concept that is also supported by the genetic association of MS with MHC class II haplotypes and of molecules involved in the regulation of MHC Class II restricted T-cell mediated inflammation (27). However, at the time, when new lesions and neurodegeneration appear in the nervous system, only sparse or even no CD4^+^ T-cells are present in the tissue (37, 57). Thus, CD4^+^ T-cells may be involved in the initiation of the immune response in MS patients, but less in the effector stage of brain inflammation, immune mediated demyelination and neurodegeneration. In contrast to many other acute of chronic inflammatory brain diseases, cells from the B-cell lineage are a major component of the adaptive immune inflammation in the brain and spinal cord of MS patients (37). They consist in the early stage and in early lesions mainly of CD20^+^ B-cells, while during lesion maturation and in the progressive stage of the disease plasma-blasts and plasma cells dominate (37, 47). Their possible role in the propagation of demyelination and neurodegeneration is indicated by the highly effective therapeutic response of MS patients in clinical trials targeting B-cells by antibodies against CD20 (58, 59). B-cells in MS lesions may augment T-cell mediated inflammation for instance through effective auto-antigen presentation, but may also have direct effects in disease pathogenesis. In this line some data suggest that B-cells within the central nervous system of MS patients produce factors that can trigger demyelination and neurodegeneration *in vitro* (60, 61). In addition, however, plasma cells in MS lesions express interleukin 10, suggesting a potential regulatory role (37).

It has been suggested that lymphocyte infiltration is less pronounced in patients with primary progressive compared to secondary progressive MS (62), but this observation was restricted to the analysis of focal white matter lesions and based on a limited number of patients only. It was not confirmed in a more recent study (47). In addition, a major component of the inflammatory response accumulates in the large Virchow Robin spaces of periventricular veins (63) and in the meninges, where they may form inflammatory aggregates, which in the most severe variants reveal the structure of tertiary lymphatic follicles (64). Some studies described a lower degree of meningeal inflammation and in particular the absence of tertiary follicle like structures in the meninges of PPMS in comparison to SPMS patients (65, 66), but this was not the case in PPMS patients with rapid disease progression in other studies (49, 67).

### Focal White Matter Lesions

The inflammatory process in MS is associated with the formation of different focal lesion types in the white matter of the brain and spinal cord. They include classical active lesions with pronounced blood brain barrier injury, chronic active or slowly expanding lesions with a low degree of demyelinating activity at the lesion edge and no major blood brain barrier damage, inactive lesions and remyelinated shadow plaques (46, 68, 69). While classical active focal white matter lesions are most numerous in patients with early disease (acute and relapsing MS; Figure 1), they become rare in the patients who have entered the progressive stage. In the latter patients slowly expanding or chronic active lesions contribute on average 30% of all focal demyelinated or remyelinated plaques [(46); Figure 3]. Their speed of expansion is very low and longitudinal follow up for several years is necessary to document their enlargement at 7T MRI (70). MRI studies indicate that focal white matter lesions are less abundant in patients with primary vs. secondary progressive MS (71, 72). However, very large neuropathological studies on more than 300 patient autopsies did not reveal significant differences between PPMS and SPMS patients in the global extent of white matter plaques or the relative incidence of different focal white matter lesions, such as active, chronic active (slowly expanding), or inactive plaques (46, 69). This discrepancy between MRI and pathology data may in part be due to a sampling bias in pathology, where the selection of tissue areas for detailed analysis is focused on brain areas with macroscopically visible lesions. In this line, a study focusing on the analysis of very large hemispheric and double hemispheric MS brain section showed a lower incidence of active white matter lesions and more remyelinated plaques in the brain of patients with PPMS compared to SPMS, but this study was based on a rather small sample of patients (73).

The issue is further complicated by the observation of a subset of MS patients, who present with a cortico/spinal variant of multiple sclerosis. In these patients focal demyelinated white matter lesions are present only in the spinal cord and are associated with extensive cortical demyelination and neurodegeneration (74). They were present in cohorts of SPMS as well as of PPMS. Such patients show diffuse mainly periventricular white matter abnormalities in the brain in MRI. The nature of these diffuse white matter abnormalities is currently unresolved, but may be due to a combination of diffuse white matter inflammation, secondary degeneration due to neuronal loss in the cortex and age related comorbidity, such as small vessel disease [leukoarayosis; (74), see Figure 1 in (67)]. An extreme variant of this scenario appears to be a condition, designated as cortical variant of MS, which appears to be due to severe cortical damage with only very sparse and small white matter lesions in the brain and spinal cord (67, 75).

### Demyelination in the Gray Matter

Cortical lesions, present in the forebrain, the cerebellum, and the hippocampus, have recently been identified as a major substrate of MS pathology [(49, 65, 66, 76–78); Figure 3]. More than 90% of cortical lesions can be visualized by post mortem scanning of the brain by high field magnetic resonance imaging using very long imaging times (79, 80). However so far, their detection in the living patients *in vivo* is very incomplete, depicting only an estimate of 10–15% of cortical demyelination, even when ultra-high field MRI is applied (81). Most lesions depicted in MRI are cortico/subcortical or intra-cortical, while the most abundant subpial lesions largely remain unrecognized. Cortical lesions, including the subpial lesions, may already arise in the early stages of MS, being present in brain biopsies or autopsies of patients with a disease duration of weeks to months (82), but the number and size increases with disease duration, thus being most extensive in patients with progressive disease (49). So far no significant differences in the incidence and size of cortical lesions have been observed between patients with primary or secondary progressive disease.

As discussed above, subpial cortical lesions are associated with meningeal inflammation (Figure 3). Meningeal inflammation is present in the form of diffuse infiltrates or of inflammatory aggregates containing densely packed T-cells, B-cells, and Plasma cells, which in most severe forms resemble tertiary lymphatic follicles (64). The severity of meningeal inflammation correlates with the extent of subpial cortical demyelination and neurodegeneration (83). Neuropathology, based on a limited number of cases, indicated that tertiary lymph follicles in the meninges are a feature of patients with secondary progressive disease (65), being absent in patients with primary progressive MS (66). However, this does not appear to be the case in PPMS patients with rapid disease progression (49). Furthermore, the presence of tertiary lymph follicles is not mandatory for active cortical demyelination, since active cortical lesions are also present in cases with lower degree and more diffuse meningeal inflammation.

While lymphocytes and plasma cells are restricted to the meninges, active cortical demyelination occurs in an outside/in gradient and is associated with microglia activation at the site of active myelin destruction (49, 84). These observations indicate that active demyelination and neurodegeneration in the cortex may be driven by a soluble factor, produced in the meningeal inflammatory infiltrates, either directly or indirectly through microglia activation (42, 84, 85). The existence of such a soluble demyelinating or neurotoxic factor has been described in the serum and cerebrospinal fluid of MS patients decades ago (86) and it seems to be produced by B-lymphocytes (60, 61). Although the nature of the demyelinating or neurotoxic factor has so far not been identified, several potential candidates have been suggested, including ceramide (87) or semaphorin 4A (88).

Cortical demyelination is accompanied by profound axonal and neuronal degeneration (50, 89). It results in profound neuronal loss following a gradient from the meningeal surface toward the depth of the cortical ribbon (84). Although neuronal loss in the cortex is highest in demyelinated lesions, it is also present in the normal appearing cortex (74).

Other gray matter areas, affected in the disease process of MS are the deep gray matter nuclei, including the thalamus, hypothalamus and basal ganglia as well as the gray matter of the spinal cord (50, 90, 91). As in the cerebral cortex these structures contain focal demyelinated plaques, but active lesions at these sites are not associated with meningeal, but perivascular inflammation. As in cortical lesions, active demyelination expands at a distance from the lymphocytic perivascular infiltrates and is associated with microglia activation. In contrast to cortical lesions, demyelinated plaques in deep gray matter nuclei are already present at early stages of MS and their number and size only moderately increases with disease duration (50). In addition to the presence of focal demyelinated lesions there is also a profound diffuse neuronal loss within the deep gray matter nuclei, associated with inflammation and oxidative injury, which may reflect augmentation of oxidative injury by the high iron content in the deep gray matter nuclei (50).

### Diffuse Injury in the Normal Appearing White and Gray Matter

Diffuse injury in the normal appearing white matter is prominent in the MS brain and spinal cord, in particular in patients in the progressive stage of the disease. It consists of small perivascular inflammatory infiltrates, some diffuse infiltration of the tissue, predominantly by CD8^+^ T-lymphocytes, diffuse axonal injury with secondary demyelination, reactive astrocytic scaring and global microglia activation. The average axonal loss in focal white matter lesions is in the range of 60% (52, 92, 93). The extent of cortical demyelination in the brain of patients with progressive MS is extensive (49) and can affect in extreme cases up to 90% of the cortical ribbon. Within the cortical lesions, but also in the surrounding normal appearing cortex, neuronal loss is seen, which may reach up to 60% of cortical nerve cells (84). Thus, a major part of the axonal neurodegeneration in the white matter appears to be due to secondary Wallerian degeneration as a consequence of axonal trans-section in plaques and neuronal loss in the gray matter (94). Wallerian tract degeneration in the human brain is a very slow process, reflected by the presence of degenerating axons even months after the focal trans-section in a lesion. Thus, ongoing axonal demise in the normal appearing white matter in the absence of lesions with active demyelination may to a major part reflect secondary anterograde or retrograde degeneration as a consequence of axonal or neuronal damage, that has occurred even months before.

Alternatively, diffuse neurodegeneration in the normal appearing gray and white matter may occur independently of focal lesions. Diffuse axonal damage in the normal appearing white matter of the spinal cord has been shown to be associated with inflammation in the meninges (95) and a similar process may trigger neuronal loss in the normal appearing cortex. In addition, age related neurodegeneration and comorbidities, such as vascular pathology and subsequent diffuse hypoxia are likely to be additional factors, driving diffuse neurodegeneration in the brain of patients with progressive MS (50).

Some studies have shown profound diffuse myelin lipid changes in the normal appearing white matter of patients with progressive MS. This can be visualized by myelin imaging in MRI as well as by neuropathological or biochemical analysis (96, 97). Overall, these changes consist of diffuse myelin abnormalities and diffuse alterations in phospholipids, and it was suggested that these changes reflect a metabolic disturbance of myelin, which may be the primary cause of MS or amplify myelin damage (98). An alternative explanation is that these changes reflect Wallerian degeneration, since they are associated with diffuse axonal injury in pathology.

All these diffuse changes in the normal appearing white and gray matter are increasing with age and disease duration of the patients and are, thus, most pronounced in patients with progressive disease. So far, however, they have been seen in similar extent in patients with PPMS and SPMS.

## Pathogenetic Implications

All the data discussed above show that there are differences in clinical disease, pathology and immunology between the relapsing and the progressive stage of MS. However, when primary and secondary progressive MS are compared with each other no qualitative differences become apparent, but there are some quantitative differences in the presence of focal and active classical white matter lesions and the global degree of inflammation, being lower in PPMS compared to SPMS. The key issue, however, is to explain the difference between early acute and relapsing MS and the progressive disease stage.

Overall these differences could be explained by acknowledging that there are two different types of inflammation in MS patients (Figures 1–3). The first, which is associated with the formation of new focal lesions mainly in the white matter, is the focal bulk invasion of inflammatory cells into the brain, which is associated with a major disturbance of the blood brain barrier. Like in experimental models of brain inflammation, such as for instance autoimmune encephalomyelitis, lymphocytes enter the brain in the course of immune surveillance, and when they recognize their cognate antigen within the central nervous system, they may become activated, produce a variety of pro-inflammatory mediators and recruit additional cells and serum components through the impaired blood brain barrier (99). It has originally been thought that this process is mediated by MHC Class II antigen restricted CD4^+^ T-lymphocytes. However, as discussed above, more recent neuropathological data and experience from therapeutic trials do not support their dominant role in patients with established disease. Instead, CD8^+^ T-cells or CD20^+^ B-cells may be more important at least at the stage, when the lesions arise or expand in the brain and spinal cord.

The mere presence of T- and B-cells in the brain of MS patients alone does not allow conclusions regarding their potential involvement in the disease process. The CD8^+^ T-cells in the MS brain show the phenotype of tissue resident memory cells. They could have entered the brain and spinal cord as disease-unrelated bystander cells during disease activity and persist as tissue resident memory cells without any direct involvement in the disease process. Support for this view comes from a recent study, showing that similar CD8^+^ tissue resident memory T-cells without signs of activation also populate in small numbers the brain of normal controls and patients with neurodegenerative disease (100). Similarly, a major component of the intrathecal antibody response in MS patients is directed against measles, rubella and varicella zoster virus (101), possibly reflecting the B-cell repertoire at the time of their recruitment into the inflammatory brain lesions. However, in contrast to controls the CD8^+^ T-cells in the MS brain focally proliferate and show signs of activation (37, 57) or clonal expansion (102), indicating local antigen recognition. Such cells could either promote disease or have regulatory function. Although they do not express interleukin 10 or TGF-ß, a regulatory function through interferon-γ of perforin mediated mechanisms, as suggested to operate in a mouse model of EAE cannot be excluded (103, 104). However, in the MS brain these cells are associated with active demyelination and neurodegeneration, indicating a disease promoting role in the lesions (37, 57). Regarding B-cells the therapeutic effect of anti-CD20 antibodies supports their pathogenetic role in MS patients. However, plasmablasts and plasma cells within the MS lesions highly express interleukin 10 (37) suggesting that these cells may ameliorate inflammation. Thus, the role of cells derived from the B-cell lineage in MS lesion may depend upon their stage of differentiation in different types or activity stages of the lesions (105, 106).

The acute inflammatory process may lead to focal areas of primary demyelination with variable axonal injury, mainly accomplished by activated microglia and macrophages and possibly also by specific antibodies and may give rise to the appearance of different types of active focal MS plaques (107). One possible pathogenic demyelinating autoantibody is directed against myelin oligodendrocyte glycoprotein (MOG), which, however, is present in patients with a disease that turned out to be different from MS (108). In addition, antibodies binding to the surface of oligodendrocytes and astrocytes (109, 110) have been found in MS patients, but the molecular nature of the target antigen is so far undefined. The acutely recruited and activated lymphocytes are in part destroyed by programed cell death (37) and microglia and macrophages are transformed in part into an anti-inflammatory phenotype (38). Thus, these lesions may become inactive and a subset of them may even be repaired by remyelination (53). New bouts of the disease (also termed disease activity in clinical terms) will then be induced by new waves of T-cells and B-cells, focally entering the brain in association with blood brain barrier damage, a process termed “disease activity” in clinical and imaging studies (2).

The second pattern of inflammation in the MS brain is an inflammatory reaction, which accumulates in the large connective tissue spaces of the brain and spinal cord, dominantly affecting the meninges (111) and the large periventricular Virchow Robin spaces (63). Clearance of T- and B-cells from the central nervous system by apoptosis is highly effective for those cells, which penetrate the brain tissue, but is only minor or absent in lymphocytes present in the perivascular and meningeal connective tissue (112). The phenotype of CD8^+^ T-cells in these chronic lesions is similar to that of tissue resident memory T-cells, which are largely present in an inactive stage, but show focal spots of activation (37, 57). Regarding cells of the B-cell lineage, CD20 positive cells are most frequent in active lesions, but the majority of cells present in chronic lesions are plasmablasts and plasma cells (37). In the meninges and perivascular space this inflammatory reaction is present diffusely but it may form focal aggregates or structures, which resemble tertiary lymph follicles with clearly separated T-cell, B-cell and plasma cells areas (111). In contrast to the inflammatory reaction in classical active white matter lesions blood brain barrier damage is minor or absent in this compartmentalized inflammatory reaction in chronic MS (48). The meningeal and perivascular infiltrates are associated with slow expansion of pre-existing focal white matter lesions, with subpial cortical demyelination and with diffuse damage of the normal appearing white and gray matter, which are the changes typically found in the brain and spinal cord of patients with active demyelination and neurodegeneration in the progressive stage of the disease (113). Tissue injury may at least in be part mediated by a cascade involving microglia and macrophage activation, oxidative injury and mitochondrial damage (5). All these data indicate that demyelination and neurodegeneration in MS is driven by the inflammatory process in all disease stages. However, it is unlikely that inflammatory T- and B-cells interact by direct contact with the specific target cells. More likely, soluble factors, produced by the inflammatory cells, may exert tissue damage either directly or indirectly by the activation of microglia or macrophages (84, 85).

These two types of inflammation occur in parallel in patients with relapsing as well as progressive disease. However, classical active plaques with inflammation and leaky blood brain barrier are most frequent in the early disease stages and then decline with age and disease duration in patients with progressive disease and are even less frequent in particular in patients with primary progressive disease (46, 69, 73). It is so far not clear, whether these two different types of inflammation reflect immune reactions to different target antigens within the brain or just represent inflammatory reactions to a single antigen. To answer this question, knowledge on the specific target antigens for T-cells and B-cells in the MS brain is required, but so far lacking (6, 7).

In summary, inflammation in the brain and spinal cord is present in all patients with active disease, reflected by classical active lesions in the early disease stages and by slowly expanding lesions in the white and gray matter and ongoing neurodegeneration in the progressive stage of the disease. The dominant inflammatory cells are CD8^+^ T-cells with proliferenation and activation in early stages of classical active lesions and a phenotype of tissue resident memory cells with focal activation in lesions with ongoing demyelination and neurodegeneration in the progressive stage. Numerous CD20^+^ B-cells are found in perivascular and meningeal inflammatory aggregates in relation to lesion activity in all disease stages, but they apparently transform into plasmablasts and plasma cells in the course of lesion maturation. Depending on the lesion stage lymphocytes may play a role in the induction of tissue damage or have regulatory function. Demyelination and neurodegeneration takes place at a distance from the T- and B-lymphocytes and is associated with activated microglia and macrophages. The structure of active lesions suggests that tissue damage is driven by a soluble factor, produced by lymphocytes. Neither the molecular nature of the soluble factor nor the antigen specificity of the infiltrating T- and B-cells has been identified so far.

## Are Different Courses of MS Reproduced in Experimental Autoimmune Encephalomyelitis?

Experimental autoimmune encephalomyelitis (EAE) is an acute or chronic neuro-inflammatory disease of the brain and spinal cord, induced by sensitization of animals with tissue or specific antigens of the central nervous system. The value and limits of different EAE models for MS research has recently been reviewed in detail (99), and therefore only few points directly related to the current topic are mentioned here. EAE can be induced in most, if not all, mammalian species including humans and leads to an inflammatory disease, which, depending upon the model, is associated with focal plaques of demyelination and/or diffuse neurodegeneration. The respective experimental models provide excellent tools to elucidate basic mechanisms of brain inflammation and immune mediated tissue injury in the central nervous system, mediated by different T-cell populations and components of the innate immune system. Most importantly, many anti-inflammatory or immunomodulatory therapies, which have been proven effective in MS patients, have been developed with the help of EAE models. However, the value of these treatments in patients, who have reached the progressive stage of MS, is limited. In addition, effective anti-inflammatory treatments in MS so far target many different immune cells simultaneously, including different T-cell populations, B-lymphocytes and in part also macrophages, while treatments selectively directed against the MHC Class II restricted CD4^+^ T-cell population, which drives inflammation in most EAE models, have so far not provided significant beneficial effects (99). Furthermore, the nature of the inflammatory response and the mechanisms of demyelination and neurodegeneration in the lesions are different between MS and EAE, and so far no EAE model is available, which reproduces the specific clinical and neuropathological features of progressive MS (99). Thus, despite the undisputed value of EAE for basic research related to mechanisms of brain inflammation and immune mediated tissue injury, their value as models for MS is limited and the elucidation of specific mechanisms related to MS pathogenesis depends on the analysis of the human disease itself. However, EAE models induced by sensitization with myelin oligodendrocyte glycoprotein (MOG) in rats and primates are perfect models for MOG auto-antibody associated inflammatory demyelinating disease (99), which however is a disease distinctly different from MS (108).

## Future Perspectives of MS Research

There are at present a number of key questions, which require focused attention:

1. One key point is to elucidate the function of tissue resident memory CD8^+^ T-cells, which are the most abundant inflammatory cells in MS lesions. Further studies are necessary to define their activation stages, their molecular profiles and their functional role in MS lesions in relation to active tissue damage, clearance of tissue debris and tissue repair. CD8^+^ resident memory cells have originally been defined and functionally characterized in experimental models of virus induced disease (114, 115). It is unlikely that such cells will develop in a condition of classical autoimmunity, when they are directed against an antigen, which is ubiquitously present within the target tissue and is not eliminated in the course of the inflammatory response. Thus, it will be of critical importance to identify the specific antigen(s), which are recognized by tissue infiltrating T- and B-lymphocytes within MS lesions at different stages of lesion formation and disease development (7).
2. Accumulating evidence supports an important role of CD20 positive B-lymphocytes in MS pathogenesis. Although their role may in part be related to the augmentation of T-cell mediated inflammatory responses (116), for instance through effective antigen presentation, an (additional) more direct involvement in the inflammatory process is likely (see above). In addition, they may have disease promoting or regulatory functions, dependent on their differentiation stage in the evolution of the brain lesions. Functional studies so far have concentrated on the production and pathogenic involvement of (auto) antibodies, but little is known regarding the role of B-cells themselves in the process of immune surveillance of the normal brain, in brain inflammation and in immune mediated tissue injury.
3. Another key feature, which is not well-reproduced in models of EAE is cortical demyelination, associated with meningeal inflammation. The only EAE models, which show MS like cortical demyelination are those, which are mediated by a combination of an encephalitogenic T-cell response in concert with a demyelinating antibody response against myelin oligodendrocyte glycoprotein [MOG, (99, 117)]. Despite extensive search the identification of MS-specific target antigens for demyelinating antibodies was not convincingly successful up to now.
4. Most evidence from pathological studies suggests that demyelination and neurodegeneration in MS is driven by the inflammatory cells, but that these processes are not directly induced by cellular contacts. In addition, plaque like primary demyelination is a specific feature of MS, not seen in other inflammatory conditions of the brain and spinal cord with the exception of diseases with viral infection of oligodendrocytes (89). Evidence from expanding cortical lesions and slowly expanding white matter lesions suggest that demyelination and neurodegeneration is driven by an MS specific soluble factor, produced by inflammatory cells, which induces tissue damage either directly or indirectly through microglia activation (83), and that this soluble factor may be produced by B-cells from MS patients, but not from controls (60). To identify the molecular nature of this soluble factor will be instrumental for our understanding of MS pathogenesis.

## Author Contributions

The author confirms being the sole contributor of this work and has approved it for publication.

### Conflict of Interest Statement

The author declares that the research was conducted in the absence of any commercial or financial relationships that could be construed as a potential conflict of interest.

## References

[1] Lassmann H Brück W Lucchinetti C. The immunopathology of multiple sclerosis: an overview. Brain Pathol. (2007) 17:210–8. 10.1111/j.1750-3639.2007.00064.x 17388952 PMC8095582

[2] Lublin FD Reingold SC Cohen JA Cutter GR Sørensen PS Thompson AJ. Defining the clinical course of multiple sclerosis. The 2013 revision. Neurology (2014) 83:1–9. 10.1212/WNL.0000000000000560 24871874 PMC4117366

[3] Trapp BD Nave KA Multiple sclerosis: an immune or neurodegenerative disorder? Ann Rev Neurosci. (2008) 31:247–2696. 10.1146/annurev.neuro.30.051606.094313 18558855

[4] Antel J Antel S Caramanos Z Arnold DL Kuhlmann T Promary progressive multiple sclerosis: part of the MS disease spectrum or separate disease entity?- Acta Neuropathol. (2012) 123:627–38. 10.1007/s00401-012-0953-0 22327362

[5] Mahad DH Trapp BD Lassmann H. Pathological mechanisms in progressive multiple sclerosis. Lancet Neurol. (2015) 14:183–93. 10.1016/S1474-4422(14)70256-X 25772897

[6] Hohlfeld R Dornmair K Meinl E Wekerle H. The search for the target antigens of multiple sclerosis, part 1: autoreactive CD4+ T lymphocytes as pathogenic effectors and therapeutic targets. Lancet Neurol. (2016) 15:198–209. 10.1016/S1474-4422(15)00334-8 26724103

[7] Hohlfeld R Dornmair K Meinl E Wekerle H. The search for the target antigens of multiple sclerosis, part 2: CD8+ T cells, B cells, and antibodies in the focus of reverse-translational research. Lancet Neurol. (2016) 15:317–31. 10.1016/S1474-4422(15)00313-0 26724102

[8] Claes N Fraussen J Stinissen P Hupperts R Somers V. B cells are multifunctional players in multiple sclerosis pathogenesis: insights from therapeutic interventions. Front Immunol. (2015) 6:642. 10.3389/fimmu.2015.00642 26734009 PMC4685142

[9] Dargahi N Katsara M Tselios T Androutsou ME de Courten M Matsoukas J. Multiple sclerosis: immunopathology and treatment update. Brain Sci. (2017) 7:E78. 10.3390/brainsci7070078 28686222 PMC5532591

[10] Rice CM Cottrell D Wilkins A Scolding NJ. Primary progressive multiple sclerosis: progress and chalenges. J Neurol Neurosurg Psychiat. (2013) 84:1100–6. 10.1136/jnnp-2012-304140 23418213

[11] Abdelhack A Weber MS Tumani H Primary progressive multiple sclerosis: putting together the puzzle. Front Neurol. (2017) 8:234 10.3389/fneur.2017.00234 28620346 PMC5449443

[12] Confafreux C Vukusic S Moreau T Adeleine P Relapses and progression of disability in multiple sclerosis. N Engl J Med. (2000) 343:1430–8. 10.1056/NEJM200011163432001 11078767

[13] Leray E Yaouang J Le Page E Coustans M Laplaud D Oger J. Evidence of a two stage disability progression in multiple sclerosis. Brain (2010) 133:1900–13. 10.1093/brain/awq076 20423930 PMC2892936

[14] Kuchling J Ramien C Bozin I Dörr J Harms L Rosche B Identical lesions morphology in primary progressive and relapsing-remitting MS. An ultrahigh filed MRI study. Mult Scler. (2014) 20:1866–71. 10.1177/1352458514531084 24781284

[15] Scalfari A Lederer C Daumer M Nicholas R Ebers GC Muraro PA. The relationship of age with the clinical phenotype in multiple sclerosis. Mult Scler. (2016) 22:1750–8. 10.1177/1352458516630396 26869531

[16] Zeydan B Kantarci OH. Progressive forms of multiple sclerosis: distinct entity of age dependent phenomena. Neurol Clin. (2018) 36:163–72. 10.1016/j.ncl.2017.08.006 29157397

[17] Ebers GC. Natural history of primary progressive multiple sclerosis. Mult Scler. (2004) 10(Suppl. 1):S8–13. 10.1191/1352458504ms1025oa 15218804

[18] Sadovnick AD. Differential effects of genetic susceptibility factors in males and females with multiple sclerosis. Clin Immunol. (2013) 149:170–5. 10.1016/j.clim.2013.05.002 23796437

[19] International Multiple Sclerosis Genetcis Consortium Analysis of immune-related loci identifies 48 new susceptibility variants for multiple sclerosis. Nat. Genet. (2013) 45:1353–60. 10.1038/ng.2770 24076602 PMC3832895

[20] International Multiple Sclerosis Genetcis Consortium Low-frequency abd rare-coding variation contributes to multiple sclerosis risk. Cell (2018) 175:1679–87.e7. 10.1016/j.cell.2018.09.049 30343897 PMC6269166

[21] Chataway J Mander A Robertson N Sawcer S Deans J Fraser M. Multiple sclerosis in sibling pairs: an analysis of 250 families. J Neurol Neurosurg Psychiatr. (2001) 71:757–61. 10.1136/jnnp.71.6.757 11723196 PMC1737661

[22] Oturai AB Ryder LP Fredrickson S Myhr KM Celius EG Harbo HF Concordance of disease course and age of onset in Scandinavian multiple sclerosis co-affected sib pairs. Mult Scler. (2004) 10:5–8. 10.1191/1352458504ms975oa 14760946

[23] Jia X Madireddy L Caillier S Santaniello A Esposito F Comi G Genomic sequencing uncovers phenocopies in primary progressive multiple sclerosis. Ann Neurol. (2017) 84:51–63. 10.1002/ana.25263 PMC6119489 29908077

[24] Pan G Simpson S van der Mei I Charlesworth JC Lucas R Ponsonby AL. Role of genetic susceptibility variants in predicting clinical course in multiple sclerosis: a cohort study. J Neuro Neurosurg Psychiat. (2016) 87:1204–11. 10.1136/jnnp-2016-313722 27559181

[25] Wang Z Sadovnick AD Traboulsee AL Ross JP Bernales CQ Encarnacion M. Nuclear receptor NR1H3 in familial multiple sclerosis. Neuron (2016) 90:948–54. 10.1016/j.neuron.2016.04.039 27253448 PMC5092154

[26] International Multiple Sclerosis Genetcis Consortium NR1H3 p.Arg415Gln is not associated to multiple sclerosis risk. Neuron(2016) 92:333–5. 10.1016/j.neuron.2016.09.052 27764667 PMC5641967

[27] Sawcer S Hellenthal G Pirinen M Spencer CC Patsopoulos NA Moutsianas L. Genetic risk and a primary role for cell-mediated immune mechanisms in multiple sclerosis. Nature (2011) 476:214–9. 10.1038/nature10251 21833088 PMC3182531

[28] Housley WJ Pitt D Hafler DA. Biomarkers in multiple sclerosis. Clin Immunol. (2015) 161:51–8. 10.1016/j.clim.2015.06.015 26143623

[29] Paul A Comabella M Gandhi R Biomarkers in multiple sclerosis. Cold Spring Harb Perspect Med. (2018) a029058 10.1101/cshperspect.a029058 PMC6396336 29500303

[30] Abdelhak A Hottenrott T Mayer C Hintereder G Zettl U Stich O CSF profile in primary progressive multiple sclerosis; reexploring the basics. PLoS ONE (2017) 12:e0182647 10.1371/journal.pone.0182647 28797088 PMC5552348

[31] Thompson AJ Reingold SC Cohen JA. Applying the 2017 McDonald diagnostic criteria for multiple sclerosis - Authors' reply. Lancet Neurol. (2018) 17:499–500. 10.1016/S1474-4422(18)30168-6 29778360

[32] Hohlfeld R. Immunologic factors in primary progressive multiple sclerosis. Mult Scler. (2004) 10:S16–22. 10.1191/1352458504ms1026oa 15218805

[33] Bielekova B Komori M Xu Q Reich DS Wu T Cerebrospinal fluid Il12p40, CXCL13 and Il-8 as a combinatorial biomarker of active intrathecal inflammation. PLoS ONE (2012) 7:e48370 10.1371/journal.pone.0048370 23226202 PMC3511462

[34] Han S Lin YC Wu T Salgado AD Mexhitaj I Wuest SC Comprehensive immunophenotyping of cerebrospinal fluid cells in patients with neuroimmunological disease. J Immunol. (2014) 192:2551–63. 10.4049/jimmunol.1302884 24510966 PMC4045479

[35] Barbour C Kosa P Komori M Tanigawa M Masvekar R Wu T Molecular-based diagnosis of multiple sclerosis and ist progressive stage. Ann Neurol. (2017) 82:795–812. 10.1002/ana.25083 29059494 PMC5743213

[36] Wurth S Kuenz B Bsteh G Ehling R DiPauli F Heegen H. Cerebrospinal fluid B cells and disease progression in multiple sclerosis - a longitudinal study. PLoS ONE (2017) 12:e0182462. 10.1371/journal.pone.0182462 28777826 PMC5544180

[37] Machado-Santos J Saji E Tröscher A Paunovic M Liblau R Gabriely G. The compartmentalized inflammatory response in the multiple sclerosis brain is composed of tissue-resident CD8+ T lymphocytes and B cells. Brain (2018) 141:2066–82. 10.1093/brain/awy151 29873694 PMC6022681

[38] Zrzavy T Hametner S Wimmer I Butovsky O Weiner H Lassmann H. Loss of ‘homeostatic' microglia and patterns of their activation in active multiple sclerosis. Brain (2017) 140:1900–13. 10.1093/brain/awx113 28541408 PMC6057548

[39] Thompson EJ Keir G. Laboratory investigation of cerebrospinal fluid proteins. Ann Clin Biochem. (1990) 27:425–35. 10.1177/000456329002700503 1704202

[40] Cepok S Zhou D Vogel F Rosche B Grummel V Sommer N. The immune response at onset and during recovery from Borrelia buf'rgdorferi meningoradiculitis. Arch Neurol. (2003) 60:849–55. 10.1001/archneur.60.6.849 12810490

[41] Cepok S Rosche B Brummel V Vogel F Zhou D Sayn J. Short-lived plasma blasts are the main B-cell effector subset during the course of multiple sclerosis. Brain (2005) 128:1667–76. 10.1093/brain/awh486 15800022

[42] Magliozzi R Howell OW Nicholas R Cruciani C Castellaro M Romualdi C. Inflammatory intrathecal profiles and cortical damage in multiple sclerosis. Ann Neurol. (2018) 83:739–55. 10.1002/ana.25197 29518260

[43] Mane-Martinez MA Olsson B Bau L Matas E Cobo-Calvo A Andreasson U. Glial and neuronal markers in cerebrospinal fluid in different types of multiple sclerosis. J Neuroimmunol. (2016) 299:112–7. [Epub ahead of print] 10.1016/j.jneuroim.2016.08.004 27725108

[44] Barro C Benkert P Disanto G Tsagkas C Amann M Naegelin Y Serum neurofilament as a predictor of disease worsening and brain and spinal cord atrophy in multiple sclerosis. Brain (2018) 141:2382–91. 10.1093/brain/awy154 29860296

[45] Stoessel D Stellmann JP Willimg A Behrens B Rosenkranz SC Hodecker SC. Metabolomic profiles for primary progressive multiple sclerosis stratification and disease course monitoring. Front Hum Neurosci. (2018) 12:226. 10.3389/fnhum.2018.00226 29915533 PMC5994544

[46] Frischer JM Weigand SD Guo Y Kale N Parisi JE Pirko I. Clinical and pathological insights into the dynamic nature of the white matter multiple sclerosis plaque. Ann Neurol. (2015) 78:710–21. 10.1002/ana.24497 26239536 PMC4623970

[47] Frischer JM Bramow S Dal Bianco A Lucchinetti CF Rauschka H Schmidbauer M. The relation between inflammation and neurodegeneration in multiple sclerosis brains. Brain (2009) 132:1175–89. 10.1093/brain/awp070 19339255 PMC2677799

[48] Hochmeister S Grundtner R Bauer J Engelhardt B Lyck R Gordon G. Dysferlin is a new marker for leaky brain blood vessels in multiple sclerosis. J Neuropathol Exp Neurol. (2006) 65:855–65. 10.1097/01.jnen.0000235119.52311.16 16957579

[49] Kutzelnigg A Lucchinetti CF Stadelmann C Bruck W Rauschka H Bergmann M. Cortical demyelination and diffuse white matter injury in multiple sclerosis. Brain (2005) 128(Pt 11):2705–12. 10.1093/brain/awh641 16230320

[50] Haider L Simeonidou C Steinberger G Hametner S Grigoriadis N Deretzi G. Multiple sclerosis deep grey matter: the relation between demyelination, neurodegeneration, inflammation and iron. J Neurol Neurosurg Psychiatry (2014) 85:1386–95. 10.1136/jnnp-2014-307712 24899728 PMC4251183

[51] Schmierer K Miquel ME. Magnetic resonance imaging correlates of neuro-axonal pathology in the MS spinal cord. Brain Pathol. (2018) 28:765–72. 10.1111/bpa.12648 30375114 PMC8028680

[52] Bjartmar C Kidd G Mork S Rudick R Trapp BD. Neurological disability correlates with spinal cord axonal loss and reduced N-acetyl aspartate in chronic multiple sclerosis patients. Ann-Neurol. (2000) 48:893–901. 10.1002/1531-8249(200012)48:6<893::AID-ANA10>3.0.CO;2-B 11117546

[53] Patrikios P Stadelmann C Kutzelnigg A Rauschka H Schmidbauer M Laursen H. Remyelination is extensive in a subset of multiple sclerosis patients. Brain (2006) 129:3165–72. 10.1093/brain/awl217 16921173

[54] Patani R Balaratnam M Vora A Reynolds R. Remyelination can be extensive in multiple sclerosis despite a long disease course. Neuropath Appl Neurobiol. (2007) 33:277–87. 10.1111/j.1365-2990.2007.00805.x 17442065

[55] Hayashi T Morimoto C Burks JS Kerr C Hauser SL. Dual-label immunocytochemistry of the active multiple sclerosis lesion: major histocompatibility complex and activation antigens. Ann Neurol. (1988) 24:523–31. 10.1002/ana.410240408 3266456

[56] Booss J Esiri MM Tourtellotte WW Mason DY. Immunohistological analysis of T lymphocyte subsets in the central nervous system in chronic progressive multiple sclerosis. J Neurol Sci. (1983) 62:219–32. 10.1016/0022-510X(83)90201-0 6607973

[57] van Nierop GP van Luijn MM Michels SS Melief MJ Janssen M Langerak AW Phenotypic and functional characterization of T cells in white matter lesions of multiple sclerosis patients. Acta Neuropathol. (2017) 134:383–401. 10.1007/s00401-017-1744-4 28624961 PMC5563341

[58] Hauser SL Waubant E Arnold DL Vollmer T Antel J Fox RJ. B-cell depletion with rituximab in relapsing-remitting multiple sclerosis. N Engl J Med. (2008) 358:676–88. 10.1056/NEJMoa0706383 18272891

[59] Montalban X Hauser SL Kappos L Arnold DL Bar-Or A Comi G. Ocrelizumab versus placebo in primary progressive multiple sclerosis. N Engl J Med. (2017) 376:209–20. 10.1056/NEJMoa1606468 28002688

[60] Lisak RP Benjamins JA Nedelkoska L Barger JL Ragheb S Fan B. Secretory products of multiple sclerosis B cells are cytotoxic to oligodendroglia *in vitro*. J Neuroimmunol. (2012) 246:85–95. 10.1016/j.jneuroim.2012.02.015 22458983

[61] Lisak RP Nedelkoska L Benjamins JA Schalk D Bealmear B Touil H. B cells from patients with multiple sclerosis induce cell death via apoptosis in neurons *in vitro*. J Neuroimmunol. (2017) 309:88–99. 10.1016/j.jneuroim.2017.05.004 28601295

[62] Revesz T Kidd D Thompson AJ Barnard RO McDonals WI. A comparison of the pathology of primary and secondary progressive multiple sclerosis. Brain (1994) 117:759–65. 10.1093/brain/117.4.759 7922463

[63] Esiri MM Gay D. Immmunological and neuropathological significance of the Virchow-Robin space. J Neurol Sci. (1990) 100:3–8. 10.1016/0022-510X(90)90004-7 2089138

[64] Serafini B Rosicarelli B Magliozzi R Stigliano E Aloisi F. Detection of ectopic B-cell follicles with germinal centers in the meninges of patients with secondary progressive multiple sclerosis. Brain Pathol. (2004) 14:164–74. 10.1111/j.1750-3639.2004.tb00049.x 15193029 PMC8095922

[65] Howell OW Reeves CA Nicholas R Carassiti D Radotra B Gentleman SM. Meningeal inflammation is widespread and linked to cortical pathology in multiple sclerosis. Brain (2011) 134(Pt 9):2755–71. 10.1093/brain/awr182 21840891

[66] Choi S Howell OW Carassiti D Magliozzi R Gveric D Muraro PA. Meningeal inflammation plays a role in the pathology of primary progressive multiple sclerosis. Brain (2012) 135:2925–37. 10.1093/brain/aws189 22907116

[67] Haider L Zrzavy T Hametner S Höftberger R Bagnato F Grabner G. The topograpy of demyelination and neurodegeneration in the multiple sclerosis brain. Brain (2016) 139:807–15. 10.1093/brain/awv398 26912645 PMC4766379

[68] Kuhlmann T Ludwin S Prat A Antel J Bruck W Lassmann H. An updated histological classification system for multiple sclerosis lesions. Acta Neuropathol. (2017) 133:13–24. 10.1007/s00401-016-1653-y 27988845

[69] Luchetti S Fransen NL van Eden CG Ramaglia V Mason M Huitinga I. Progressive multiple sclerosis patients show substantial lesion activity that correlates with clinical disease severity and sex. A retrospective autopsy cohort analysis. Acta Neuropathol. (2018) 135:511–28. 10.1007/s00401-018-1818-y 29441412 PMC5978927

[70] Dal-Bianco A Grabner G Kronnerwetter C Weber M Höftberger R Berger T. Slow expansion of multiple sclerosis iron rim lesions: pathology and 7 T magnetic resonance imaging. Acta Neuropathol. (2017) 133:25–42. 10.1007/s00401-016-1636-z 27796537 PMC5209400

[71] Thompson AJ Kermode AG Wicks D MacManus DG Kendall BE Kingsley DP. Major differences in the dynamics of primary and secondary progressive multiple sclerosis. Ann Neurol. (1991) 29:53–62. 10.1002/ana.410290111 1996879

[72] Thompson AJ Polamn CH Miller DH McDonals WI Brochet B Filippi M. Primary progressive multiple sclerosis. Brain (1997) 120:1085–96. 10.1093/brain/120.6.1085 9217691

[73] Bramow S Frischer JM Lassmann H Koch-Henriksen N Lucchinetti CF Sørensen PS. Demyelination versus remyelination in progressive multiple sclerosis. Brain (2010) 133:2983–98. 10.1093/brain/awq250 20855416

[74] Trapp DB Vignos M Dudman J Chang A Fisher E Staugaitis SM. Cortical neuronal densities and cerebral white matter demyelination in multiple sclerosis: a retrospective study. Lancet Neurol. (2018) 17:870–84. 10.1016/S1474-4422(18)30245-X 30143361 PMC6197820

[75] Zarei M Chandran S Compston A Hodges J. Cognitive presentation of multiple sclerosis: evidence for a cortical variant. J Neurol Neurosurg Psychiatr. (2003) 74:872–7. 10.1136/jnnp.74.7.872 12810770 PMC1738531

[76] Kutzelnigg A Faber-Rod JC Bauer J Lucchinetti CF Sorensen PS Laursen H. Widespread demyelination in the cerebellar cortex in multiple sclerosis. Brain Pathol. (2007) 17:38–44. 10.1111/j.1750-3639.2006.00041.x 17493036 PMC8095596

[77] Howell OW Schulz-Trieglaff EK Carassiti D Gentleman SM Nicholas R Roncaroli F. Extensive grey matter pathology in the cerebellum in multiple sclerosis is linked to inflammation in the subarachnoid space. Neuropath Appl Neurobiol. (2015) 41:798–813. 10.1111/nan.12199 25421634

[78] Geurts JJ Bo L Roosendaal SD Hazes T Daniels R Barkhof F. Extensive hippocampal demyelination in multiple sclerosis. J Neuropath Exp Neurol. (2007) 66:819–27. 10.1097/nen.0b013e3181461f54 17805012

[79] Pitt D Boster A Pei W Wohleb E Jasne A Zachariah CR. Imaging cortical lesions in multiple sclerosis with ultra-high-filed magnetic resonance imaging. Arch Neurol. (2010) 67:812–8. 10.1001/archneurol.2010.148 20625086

[80] Schmierer K Parkes HG So PW So PW Brandner A Ordidge RJ. High field (9.4 Tesla) magnetic resonance imaging of cortical grey matter lesions in multiple sclerosis. Brain (2010) 133:858–67. 10.1093/brain/awp335 20123726

[81] Hametner S Bianco AD Trattnig S Lassmann H Iron related changes in MS lesions and their validity to characterize MS lesion types and dynamics with ultra-high filed magnetic resonance imaging. Brain Pathol. (2018) 28:743–9. 10.1111/bpa.12643 30020556 PMC8028547

[82] Lucchinetti CF Popescu BF Bunyan RF Moll NM Roemer SF Lassmann H. Inflammatory cortical demyelination in early multiple sclerosis. N Engl J Med. (2011) 365:2188–97. 10.1056/NEJMoa1100648 22150037 PMC3282172

[83] Magliozzi R Howell O Vora A. Meningeal B-cell follicles in secondary progressive multiple sclerosis associate with early onset of disease and severe cortical pathology. Brain (2007) 130:1089–104. 10.1093/brain/awm038 17438020

[84] Magliozzi R Howell OW Reeves C Roncaroli F Nicholas R Serafini B. A Gradient of neuronal loss and meningeal inflammation in multiple sclerosis. Ann Neurol. (2010) 68:477–93. 10.1002/ana.22230 20976767

[85] Kutzelnigg A Lassmann H. Cortical demyelination in multiple sclerosis: a substrate for cognitive deficits ? J Neurol Sci. (2006) 245:123–6. 10.1016/j.jns.2005.09.021 16650874

[86] Bornstein MB Appel SH. Tissue culture studies of demyelination. Ann N Y Acad Sci. (1965) 122:280–6. 10.1111/j.1749-6632.1965.tb20212.x 14313486

[87] Vidaurre OG Haines JD Katz Sand I Adula KP Huynh JL McGraw CA Cerebrospinal fluid ceramides from patents with multiple sclerosis impair neuronal bioenergetics. Brain (2014) 137:2271–86. 10.1093/brain/awu139 24893707 PMC4164163

[88] Chiou B Lucassen E Sather M Kallianpur A Connor J Semaphorin 4A and H-Ferritin utilize Tim-1 on human oligodendrocytes: a novel neuro-immune axis. Glia (2018) 66:1317–30. 10.1002/glia.23313 29457657 PMC7009020

[89] Fischer MT Wimmer I Hoftberger R Gerlach S Haider L Zrzavy T. Disease-specific molecular events in cortical multiple sclerosis lesions. Brain (2013) 136:1799–815. 10.1093/brain/awt110 23687122 PMC3673462

[90] Vercellino M Masera S Lorenzatti M Condello C Merola A Mattioda A Demyelination, inflammation, and neurodegeneration in multiple sclerosis deep grey matter. J Neuropath Exp Neurol. (2009) 68:489–502. 10.1097/NEN.0b013e3181a19a5a 19525897

[91] Huitinga I De Groot CJ van der Valk P Kamphorst W Tilders FJ Swaab DF. Hypothalamic lesions in multiple sclerosis. J Neuropath Exp Neurol. (2001) 60:1208–18. 10.1093/jnen/60.12.1208 11764093

[92] Mews I Bergmann M Bunkowski S Gullotta F Brück W. Oligodendrocyte and axon pathology in clinically silent multiple sclerosis lesions. Mult Scler. (1998) 4:55–62. 10.1177/135245859800400203 9599334

[93] Kornek B Storch M Weissert R Wallstroem E Stefferl A Olsson T. Multiple sclerosis and chronic autoimmune encephalomyelitis: a comparative quantitative study of axonal injury in active, inactive and remyelinated lesions. Amer J Pathol. (2000) 157:267–76. 10.1016/S0002-9440(10)64537-3 10880396 PMC1850217

[94] Singh S Dallenga T Winkler A Roemer S Maruschak B Siebert H. Relationship of acute axonal damage, Wallerian degeneration and clinical disability in multiple sclerosis. J Neurinflammation (2017) 14:57. 10.1186/s12974-017-0831-8 28302146 PMC5356322

[95] Androdias G Reynolds R Chanal M Ritleng C Confavreux C Nataf S. Meningeal T cells associate with diffuse axonal loss in multiple sclerosis spinal cords. Ann Neurol. (2010) 68:465–76. 10.1002/ana.22054 20687208

[96] Moore GR Laule C Mackay A Leung E Li DK Zhao G. Dirty-appearing white matter in multiple sclerosis: preliminary observations of myelin phospholipid and axonal loss. J Neurol. (2008) 255:1802–11. 10.1007/s00415-008-0002-z 18821049

[97] Laule C Vavasour IM Leung E Li DK Kozlowski P Traboulsee AL. Pathological basis of diffusely abnormal white matter: insights from magnetic resonance imaging and histology. Mult Scler. (2011) 17:144–50. 10.1177/1352458510384008 20965961

[98] Laule C Pavlova V Leung E Zhao G MacKay AL Kozlowski P. Diffusely abnormal white matter in multiple sclerosis; further histologic studies provide evidence for a primary lipid abnormality with neurodegeneration. J Neuropath Exp Neurol. (2013) 72:42–52. 10.1097/NEN.0b013e31827bced3 23242281

[99] Lassmann H Bradl M. Multiple sclerosis: experimental models and reality. Acta Neuropathol. (2017) 133:223–44. 10.1007/s00401-016-1631-4 27766432 PMC5250666

[100] Smolders J Heutinck KM Fransen NL Remmerswaal EBM Hombrink P Ten Berge IJM. Tissue-resident memory T cells populate the human brain. Nat Commun. (2018) 9:4593. 10.1038/s41467-018-07053-9 30389931 PMC6214977

[101] Jarius S Eichhorn P Franciotta D Petereit HF Akman-Demir G Wich M The MRZ reaction as a highly specific marker of multiple sclerosis: re-evaluation and structures review of the literature. J Neurol. (2017) 264:453–66. 10.1007/s00415-016-8360-4 28005176

[102] Babbe H Roers A Waisman A Lassmann H Goebels N Hohlfeld R. Clonal expansions of CD8(+) T cells dominate the T cell infiltrate in active multiple sclerosis lesions as shown by micromanipulation and single cell polymerase chain reaction. J Exp Med. (2000) 192:393–404. 10.1084/jem.192.3.393 10934227 PMC2193223

[103] Sinha S Boyden AW Itani FR Crawford MP Karandikar NJ CD8(+) T-cells as immune regulators pf multiple sclerosis. Front Immunol. (2015) 6:619 10.3389/fimmu.2015.00619 26697014 PMC4674574

[104] Boyden AW Brate AA Karandikar NJ Early IFNg-mediated and late perforin mediated suppression of pathogenic CD4 T cell responses are both required for inhibition of demyelinating disease by CNS-specific autoregulatory CD8 cells. Front Immunol. (2018) 9:2336 10.3389/fimmu.2018.02336 30356717 PMC6189364

[105] Lehmann-Horn K Kinzel S Weber MS. Deciphering the role of B cells in multiple sclerosis. Towards specific targeting of pathogenic functions. Int J Mol Sci. (2017) 18:E2048. 10.3390/ijms18102048 28946620 PMC5666730

[106] Li R Patterson KR Bar-Or A. Reassessing B cell contributions in multiple sclerosis. Nat Immunol. (2018) 19:696–707. 10.1038/s41590-018-0135-x 29925992

[107] Lucchinetti C Brück W Parisi J Scheithauer B Rodriguez M Lassmann H. Heterogeneity of multiple sclerosis lesions: implications for the pathogenesis of demyelination. Ann Neurol. (2000) 47:707–17. 10.1002/1531-8249(200006)47:6<707::AID-ANA3>3.0.CO;2-Q 10852536

[108] Jarius S Ruprecht K Kleiter I Borisow N Asgari N Pitarokoili K. MOG-IgG in NMO and related disorders: a multicenter study of 50 patients. Part 2: Epidemiology, clinical presentation, radiological and laboratory features, treatment responses, and long-term outcome. J Neuroinflammation (2016) 13:280. 10.1186/s12974-016-0718-0 27793206 PMC5086042

[109] Lily O Palace J Vincent A Serum antibodies to sell surface determinants in multiple sclerosis: a flow cytometric study. Brain (2004) 127:269–79. 10.1093/brain/awh031 14662514

[110] Blauth K Soltys J Matschulat A Reiter CR Ritchie A Baird NL. Antibodies produced by clonally expanded plasma cells in multiple sclerosis cerebrospinal fluid cause demyelination of spinal cord explants. Acta Neuropathol. (2015) 130:765–81. 10.1007/s00401-015-1500-6 26511623 PMC4655138

[111] Aloisi F Pujol-Borrell R. Lymphoid neogenesis in chronic inflammatory diseases. Nat Rev Immunol. (2006) 6:205–17. 10.1038/nri1786 16498451

[112] Schmied M Breitschopf H Gold R Zischler H Rothe G Wekerle H. Apoptosis of T lymphocytes in experimental autoimmune encephalomyelitis. Evidence for programmed cell death as a mechanism to control inflammation in the brain. Am J Pathol. (1993) 143:446–52. 8342595 PMC1887018

[113] Lassmann H van Horssen J Mahad D. Progressive multiple sclerosis: pathology and pathogenesis. Nat Rev Neurol. (2012) 8:647–56. 10.1038/nrneurol.2012.168 23007702

[114] Schenkel JM Masopust D. Tissue-resident memory T cells. Immunity (2014) 41:886–97. 10.1016/j.immuni.2014.12.007 25526304 PMC4276131

[115] Steinbach K Vincenti I Kreutzfeldt M Page N Muschaweckh A Wagner I. Brain-resident memory T cells represent an autonomous cytotoxic barrier to viral infection. J Exp Med. (2016) 213:1571–87. 10.1084/jem.20151916 27377586 PMC4986533

[116] Hauser SL. The Charcot Lecture / Beating MS: a story of B cells, with twists and turns. Mult Scler J. (2015) 21:8–21. 10.1177/1352458514561911 25480864 PMC4580247

[117] Storch MK Bauer J Linington C Olsson T Weissert R Lassmann H. Cortical demyelination can be modeled in specific rat models of autoimmune encephalomyelitis and is major histocompatibility complex (MHC) haplotype-related. J Neuropathol Exp Neurol. (2006) 65:1137–42. 10.1097/01.jnen.0000248547.13176.9d 17146287

